# Rural Health Disparities in Alzheimer’s Disease and Related Dementias Biomarker Testing

**DOI:** 10.1093/geroni/igaf122.024

**Published:** 2025-12-31

**Authors:** Rashelle Hoffman, Elise Choquette, Erin Blocker, Blake Murphy, Bryant Howren, Eric Vidoni

**Affiliations:** Creighton University, Omaha, Nebraska, United States; Creighton University, Omaha, Nebraska, United States; Emporia State University, Emporia, Kansas, United States; Creighton University, Omaha, Nebraska, United States; VA Office of Rural Health, Iowa City, Iowa, United States; University of Kansas Medical Center, Kansas City, Kansas, United States

## Abstract

Early diagnosis of cognitive impairment through clinical tests and Alzheimer’s disease and related dementias (ADRD) biomarkers improves psychosocial care, reduces caregiver stress, and lowers healthcare costs. With the development of plasma biomarkers, ADRD testing has become more accessible for rural providers. However, it is unclear if rural providers have integrated these biomarkers into practice and whether barriers remain. At the 2024 Nebraska Rural Health and Vulnerable Population Conferences, 25 participants from 12 counties in Nebraska and Iowa completed a questionnaire on ADRD biomarker use and perceptions of an ADRD diagnosis. Most (64%) were patient-facing providers, while 36% were healthcare administrators. Although all providers reported regular cognitive screening in their practice, 75% were not ordering biomarker testing, with 68.75% expressing no interest. Among those using biomarkers (25%), blood plasma and PET imaging were the only methods reported. Most providers (62%) felt unqualified to order biomarkers. Across both administrators and providers, 39.1% reported that their organizations were not supportive, with 26.1% reporting only slight support for biomarker use. After a 30-minute educational session, participants showed greater positivity toward an ADRD diagnosis and increased confidence in discussing early detection. While cognitive screening is common in rural settings, there is limited use of biomarker testing, primarily due to a lack of knowledge about ADRD biomarker practices.

